# Acute Rhinosinusitis in Children with Inhalant Allergies

**DOI:** 10.3390/children9060836

**Published:** 2022-06-05

**Authors:** Aleksandra Pietraś, Grażyna Mielnik-Niedzielska

**Affiliations:** Chair and Department of Pediatric Otolaryngology, Phoniatrics and Audiology, Medical University of Lublin, 20-093 Lublin, Poland; grazyna.niedzielska@wp.pl

**Keywords:** inhalant allergy, acute rhinosinusitis, pediatric population

## Abstract

Rhinosinusitis is an essential medical problem in pediatric populations. Due to a lack of studies considering allergy impact on pediatric rhinosinusitis, it seems legitimate to investigate this subject. The aim of this paper was to assess the influence of inhalant allergy on acute rhinosinusitis in children. The study involved 100 pediatric patients aged between 3 and 17 years who were admitted to the Chair and Department of Pediatric Otolaryngology, Phoniatrics and Audiology of the Medical University of Lublin due to acute rhinosinusitis. The control group consisted of 50 children without allergy, and the study group consisted of 50 children suffering from inhalant allergy. The methodology employed in this study involved medical history and laryngological examination, as well as laboratory and radiological testing. Dust mite allergy was the most common allergy among patients in the study group. Patients with allergies presented at the hospital later than patients without allergy, and their hospitalization lasted longer due to more severe sinus disease, higher inflammatory parameters, multiple sinus involvement, more frequent fever or rhinosinusitis complications, especially orbital occurrence. Most children in the control group required only pharmacological treatment. Inhalant allergy, especially dust mite allergy, contributes to more severe acute rhinosinusitis in children.

## 1. Introduction

Rhinosinusitis is an essential medical problem in pediatric populations [1]. It negatively affects quality of life and exacerbates chronic conditions, and its treatment is costly.

Rhinosinusitis in children is defined as inflammation of the nose and paranasal sinus cavities and mucosa, which contributes to as least two of mentioned symptoms: nasal congestion, postnasal drip, rhinorrhea, face pain and cough. It is necessary to observe either nasal obstruction or runny nose. Endoscopic assessment shows purulent discharge and/or mucosal oedema; polyps are rare in children. Radiological imagining (especially computed tomography) could reveal mucosal changes in the ostiomeatal complex, the lateral wall of the nasal cavity and sinuses walls [2].

Rhinosinusitis is estimated to account for 5–13% of all upper respiratory tract infections [3], with higher rates during the winter season [2]. School children may suffer from rhinosinusitis as many as 10 times per year [4]. The most frequently engaged sinuses in children include maxillary (99%) and ethmoid sinuses (91%) [5].

There are many conflicting study results concerning allergy impact on acute rhinosinusitis [6,7,8]. The European Position Paper on Rhinosinusitis and Nasal Polyps 2020 suggests the need for more thorough analysis of this matter. Due to a lack of studies considering allergy impact on pediatric rhinosinusitis, it seems legitimate to investigate this subject.

The aim of this paper was to assess the influence of inhalant allergy on acute rhinosinusitis in children admitted to the Chair and Department of Pediatric Otolaryngology, Phoniatrics and Audiology of the Medical University of Lublin.

## 2. Materials and Methods

The study was approved by Medical University of Lublin Ethics Committee on 25 November 2018 (KE-0254/261/2018). It involved 100 pediatric patients aged between 3 and 17 years who were hospitalized in our clinic between 2018 and 2020 due to acute rhinosinusitis. The control group consisted of 50 children without allergy, and the study group consisted of 50 children suffering from inhalant allergy.

The methodology of the study involved thorough medical history and full laryngological examination, as well as laboratory and radiological testing. The severity of rhinosinusitis was assessed using the Visual Analog Scale (VAS).

The natural course of acute rhinosinusitis in the analyzed groups was divided into 3 types:

1. Acute onset with fever and severe pain;
2. Subacute onset with increase in symptoms after 5 days;
3. Mild persistent symptoms after 10 days.

A paranasal sinus X-ray in occipitomental view was performed on every patient in the emergency department. C-reactive protein (CPR) levels, complete blood count and specific IgE antibody levels were assessed from serum. Nasal computed tomography was performed in the case of suspicion of complications or prior to surgery. All patients received pharmacological treatment; if no improvement was observed, a maxillary sinus puncture was performed under local anesthesia. Purulent discharge from the procedure was sent for microbiological testing. In severe cases and when rhinosinusitis complications were present, sinus surgery was performed under general anesthesia, and microbiological and histopathological tests were requested.

Based on the result of specific IgE antibody levels, we divided the study patients between study and control groups. Patients with IgE antibody levels > 0.15 kU/L towards at least one allergen were qualified for the study group, and patients whose results did not reach that level were admitted to the control group.

We compared the natural course of acute rhinosinusitis, laboratory and radiological findings, and treatment and complications between both groups.

Children were discharged from hospital when symptoms subsided and inflammation parameters normalized, with recommendations for continuation of treatment and a laryngological follow-up appointment.

Obtained data were collected in Microsoft Excel sheet and statistically analyzed with Statistica software. Statistically significant outcomes were determined by *p* value < 0.05.

## 3. Results

The study involved 100 children ages 3 to 17 years old. The mean age of participants was 8.33 ± 4.17 years old.

The IgE antibody levels of the study group were in the range of 11.6 to 3971.0 IU/mL, whereas mean levels were set at 722.1 IU/mL. Perennial allergy was observed in most patients in the study group (39 patients; 78%), and seasonal allergy was found in 11 children (22%).

The most frequently occurring allergens included house dust mite (*Dermatophagoides farinae*) (14 children) and *Dermatophagoides pteronussinus* (11 children). Five patients were allergic to *Alternaria* spp. and dog hair, four participants showed hypersensitivity towards birch and cat hair and three children were allergic to grass pollen and alder. One patient was allergic to rye.

**Figure 1:**
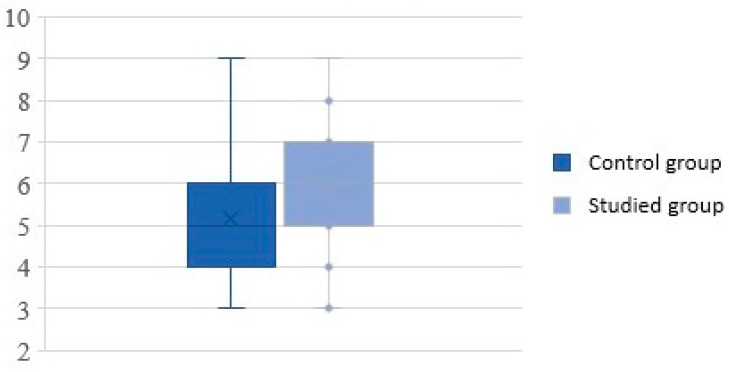
Severity of rhinosinusitis on the visual analog scale.

Subjective assessment of symptoms on the VAS scale in study group was in the range of 3 to 9, with a mean score of 5.86 ± 1.47, whereas in the control group, the VAS score was also in the range of 3 to 9 but with a mean score of 5.16 ± 1.50. Statistical analysis showed significant higher VAS scores in the study group (*p* = 0.021) (Figure 1).

Acute onset of rhinosinusitis was observed in 1 patient, subacute onset in 26 patients and mild persistent symptoms in 23 patients in the study group. Acute onset was observed in 7 patients, subacute onset in 29 patients and persistent symptoms in 14 patients in the control group. Statistical analysis revealed a significant difference in the natural course of acute rhinosinusitis in the studied groups; children with allergies presented mild persistent symptoms after 10 days more frequently, whereas children without allergies were more likely to suffer from acute onset with fever and severe pain (*p* = 0.03).

**Figure 2:**
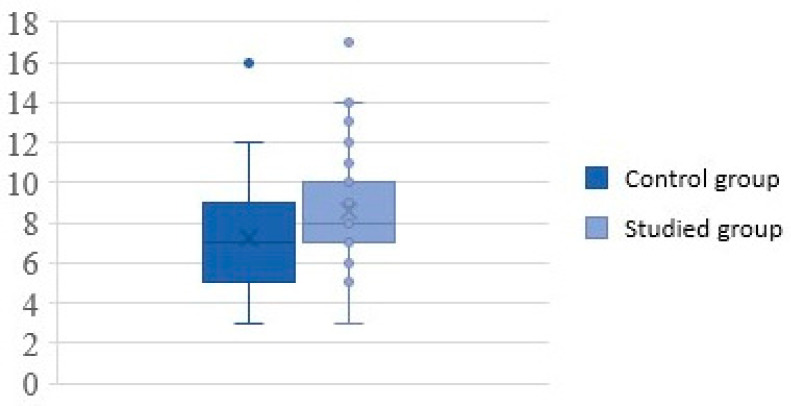
Duration of hospitalization (in days).

Study group participants spent between 3 and 17 days in the hospital ward (mean time of hospitalization was 9 ± 3 days), whereas the control group participants required between 3 and 16 in hospital, with a mean time of 7 ± 3 days. This 2-day difference was statistically significant (*p* = 0.02) (Figure 2).

Involvement of sinuses other than the maxillary sinuses was present in 33 (66%) patients in the study group and only 21 (42%) patients in the control group. Analysis showed that the difference was significant (*p* = 0.02). Moreover, ethmoid and sphenoid sinuses were more likely to be involved in children with inhalant allergies (Table 1).

Fever was present in 35 (70%) cases in the study group and in 23 (46%) cases in the control group. Fever was significantly more frequently observed in the study group compared to the control group (*p* = 0.02).

**Figure 3:**
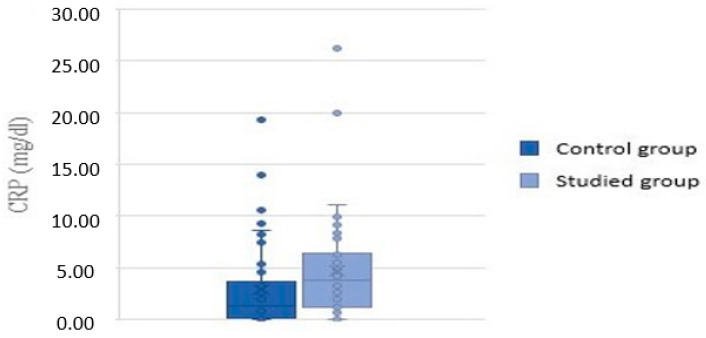
CRP values in the control and study groups.

C-reactive protein (CRP) levels measured in the study group were in the range of 0.03 to 26.25 mg/dL, with a mean value of 4.66 mg/dL. CRP levels measured in the control group were in the range of 0.03 to 19.31 mg/dL, with a mean value of 2.93 mg/dL. Analysis showed significantly higher levels of CRP in children with inhalant allergy (*p* = 0.01) (Figure 3).

In both groups, only one patient was diagnosed with meningitis. Orbital complications were diagnosed in 26 (52%) children with allergy and only 11 (22%) children without allergy (*p* = 0.002). The most common orbital complication was preseptal cellulitis, occurring twice as often in children in the study group compared to those in the control group.

There were 13 (26%) children treated effectively with pharmacotherapy, whereas 9 (18%) patients required surgery in the study group. Pharmacological treatment was effective in almost a half of patients in the control group (24 cases; 48%), and only three patients required surgery (6%).

Surgical intervention was significantly more frequently required in patients with allergy and almost twice more likely to adequately treat children with pharmacology in the control group (*p* = 0.02).

The studied variables mentioned above are presented in Table 2.

## 4. Discussion

House dust mites are organisms inextricably linked to the human environment, representing one of the most recognizable perennial allergen sources found in households, in addition to representing an important cause of allergic rhinitis [9]. It is estimated that among highly developed European populations, the frequency of allergic rhinitis could reach up to 20% [10]. House dust mite allergen is a 1–10 µm particle with enzymatic properties, which can penetrate mucosal the barrier of the respiratory tract and contribute to the development of local inflammation [11].

A study performed in China in 2018 showed that among 207 pediatric patients with acute rhinosinusitis, 44.4% suffered from inhalant allergies, among which 40.6% were perennial [12]. Similar research conducted in Thailand in 2015 revealed that 35.1% of analyzed children were allergic to inhalant particles [3]. The most common allergens included house dust mites (78.8–84.8%), grass pollen (24.2–27.3%), household animal hair (1.2–27.3%) and fungi (6.1%).

House dust mite allergy was the most frequently observed allergies in our study group, observed in 50% of patients. Our results confirm that acute rhinosinusitis is mostly accompanied by house dust mite allergy. Allergy to animal hair was diagnosed in 18% of studied cases, which corresponds to the resulted reported in the aforementioned Thai study.

A study performed by Lin at al. suggests that in pediatric populations, the presence of atopic diseases favors acute rhinosinusitis development [13]. Interleukins 4 and 13, which are involved in Th2-dependent immunological reactions, ease microorganism adhesion to respiratory tract mucosa [14].

Microbiological assessment of nose swabs taken from patients with seasonal allergic rhinitis demonstrated greater diversification when performed during pollination season, whereas the respiratory tract microbiome beyond that period was less diversified and did not differ much from bacterial swabs taken from the healthy group [15,16]. It is possible that greater colonization of nasal cavities during seasonal allergic rhinitis exacerbation contributes to a higher prevalence of upper respiratory tract infections due to the virulence of opportunistic bacteria.

Patients with inhalant allergy presented at hospital later, and their hospitalization lasted an average of 2 days longer than those without allergy. Elongated duration of acute rhinosinusitis might be a caused by the resemblance of symptoms between allergic rhinitis and acute rhinosinusitis; patients and/or chaperones might have postponed medical appointments due to an initially mild course of infection.

Elongated hospitalization in the study group might be a result of more developed inflammation (higher levels of CRP) and an advanced clinical image, including pansinusitis, fever and complications, mostly ocular. Fever forces implementation of more aggressive treatment, especially when other symptoms are very intensive, strongly suggests a bacterial etiology of acute rhinosinusitis [17].

Patients without allergy were more often treated conservatively, mainly because rhinosinusitis was limited and more responsive to medications compared to study group, in which inflammation was triggered by both infectious and allergic factors.

Radiological imaging in children with allergic rhinitis showed that mucosal oedema is usually localized in central regions of the nasal cavity and paranasal sinuses, including upper and medial nasal turbinates and the upper posterior part of the nasal septum. Inflammation can be observed in ethmoid sinuses and maxillary sinuses meatus [18]. Taking that into consideration, allergic inflammation conduces mucus stasis in ethmoid cells and infection development in this region. Our study revealed increased involvement of ethmoid cells in children with inhalant allergy, which may be a result of the abovementioned specific course of inflammation spreading in the nasal cavity and paranasal sinuses.

Sphenoid sinus meatus is situated in the nasal cavity ceiling, sharing drainage with posterior ethmoid cells via the sphenoethmoidal recess to the superior meatus of the nasal cavity [19]. Mucosal oedema (allergic and infectious) of that limited space fosters pathological secretion cumulation in sphenoid sinuses, which can be observed in radiological findings. Our study results showed that pathological changes in sphenoid sinuses in children with allergy were present two times more frequently compared to children without inhalant allergies.

It is estimated that acute rhinosinusitis complications in children are diagnosed in 5% of all cases [20]. Results of our research showed that children with allergies suffered more than twice as often as children in the control group. Research performed in the United States of America revealed that ocular complications (78%) were among the most often observed complications, whereas meningitis was diagnosed in 11% of studied cases [21]. The frequency of ocular complications in our study reached 95%; meningitis was observed in 5% of all cases. Results confirmed that ocular complication of acute rhinosinusitis in children are far more common.

Ocular complications of acute rhinosinusitis are present more often in child populations compared to adults [22,23,24,25]. The most vulnerable patients are children under 5 years of age as a result of specific anatomical factors, especially incomplete separation between orbital cavity contents and ethmoid sinuses, incomplete adhesion between facial bones and increased porosity of bone structures, which contribute to easier spread of inflammation processes between surrounding tissues [26].

Our study results suggest increased frequency of acute rhinosinusitis complications (especially ocular) in patients with inhalant allergies. In the study group, preseptal cellulitis was observed twice as often as in the control group, which may be caused by either conjunctivitis and concomitant allergic inflammation or a more advanced, prolonged stage of infectious rhinosinusitis, with the possibility of uninhibited spreading among adjoin structures.

## 5. Conclusions

Rhinosinusitis is one of the most common diseases treated with antibiotics. Creating adequate treatment algorithms and spreading knowledge about processes that contribute to this disease could minimize the excessive use of antibiotics worldwide [27].

Rhinosinusitis negatively affects quality of life and contributes to the exacerbation of chronic conditions, and its treatment is costly. Inhalant allergy, especially dust mite allergy, contributes to more severe acute rhinosinusitis in children.

## Figures and Tables

**Table 1:** Sinus involvement (apart from maxillary sinuses).

Paranasal Sinuses	Study Group	Control Group	Statistical Analysis
Ethmoid	26 (52%)	15 (30%)	*p* = 0.025
Frontal	20 (40%)	13 (26%)	*p* > 0.05
Sphenoid	16 (32%)	7 (14%)	*p* = 0.032

**Table 2:** Significant research outcomes.

Studied Factor	Control Group	Study Group	*p* Value
VAS score	3–9	3–9	0.021
	5.86 ± 1.47	5.16 ± 1.50	
Duration of hospitalization	3–16 days	3–16 days	0.02
	7 ± 3 days	9 ± 3 days	
C-reactive protein	0.03–19.31 mg/dL	0.03–26.25 mg/dL	0.01
	mean: 2.93 mg/dL	mean: 4.66 mg/dL	

## Data Availability

Raw data were generated at the Medical University of Lublin. Derived data supporting the findings of this study are available from the corresponding author (A.P.) upon request.
